# A Novel Class of Norovirus Inhibitors Targeting the Viral Protease with Potent Antiviral Activity In Vitro and In Vivo

**DOI:** 10.3390/v13091852

**Published:** 2021-09-16

**Authors:** Jana Van Dycke, Wenhao Dai, Zoe Stylianidou, Jian Li, Arno Cuvry, Emma Roux, Bingqian Li, Jasper Rymenants, Lindsey Bervoets, Peter de Witte, Hong Liu, Johan Neyts, Joana Rocha-Pereira

**Affiliations:** 1 Laboratory of Virology and Chemotherapy, KU Leuven–Department of Microbiology, Immunology and Transplantation, Rega Institute, 3000 Leuven, Belgium; Jana.vandycke@kuleuven.be (J.V.D.); zstylianidou@hotmail.com (Z.S.); Arno.cuvry@kuleuven.be (A.C.); emma.roux@kuleuven.be (E.R.); jasper.rymenants@kuleuven.be (J.R.); Lindsey.bervoets@kuleuven.be (L.B.); johan.neyts@kuleuven.be (J.N.); 2 State Key Laboratory of Drug Research, Shanghai Institute of Materia Medica, Chinese Academy of Sciences, 555 Zu Chong Zhi Road, Shanghai 201203, China; dwh1992@163.com (W.D.); jianl@simm.ac.cn (J.L.); sissilibingqian@gmail.com (B.L.); 3 College of Pharmacy, Nanjing University of Chinese Medicine, 138 Xianlin Avenue, Qixia District, Nanjing 210023, China; 4 Laboratory for Molecular Biodiscovery, KU Leuven–Department of Pharmaceutical and Pharmacological Sciences, 3000 Leuven, Belgium; Peter.dewitte@kuleuven.be

**Keywords:** Caliciviridae, antivirals, small molecule, danio rerio, infection

## Abstract

Human noroviruses (HuNoVs) are the most common cause of viral gastroenteritis resulting annually in ~219,000 deaths and a societal cost of ~USD 60 billion, and no antivirals or vaccines are available. Here, we assess the anti-norovirus activity of new peptidomimetic aldehydes related to the protease inhibitor rupintrivir. The early hit compound **4** inhibited the replication of murine norovirus (MNV) and the HuNoV GI.1 replicon in vitro (EC_50_ ~1 µM) and swiftly cleared the HuNoV GI.1 replicon from the cells. Compound **4** still inhibits the proteolytic activity. We selected a resistant GI.1 replicon, with a mutation (I109V) in a highly conserved region of the viral protease, conferring a low yield of resistance against compound **4** and rupintrivir. After testing new derivatives, compound **10d** was the most potent (EC_50_ nanomolar range). Molecular docking indicated that the aldehyde group of compounds **4** and **10d** bind with Cys139 in the HuNoV 3CL protease by a covalent linkage. Finally, compound **10d** inhibited the replication of HuNoV GII.4 in infected zebrafish larvae, and PK studies in mice showed an adequate profile.

## 1. Introduction

Human norovirus (HuNoV) infections remain a major worldwide cause of gastroenteritis that affects people of all ages. HuNoV infections can be particularly severe in children, the elderly and immunocompromised. Annually, ~700 million people will acquire a HuNoV infection and ~219,000 people die. Moreover, there is a considerable yearly economic burden of USD 60 billion linked to HuNoV infections [1]. HuNoV is very contagious and causes large outbreaks, which have a significant health and economic impact, mostly in hospital wards and nursing homes. Chronic norovirus infections are problematic in immunodeficient patients, who may have diarrhea for months. There is still no approved antiviral available to treat and/or prevent a HuNoV infection, and the current treatment is merely supportive.

The norovirus nonstructural proteins constitute an attractive target for antiviral drug design. Here we focus on the norovirus protease, which displays a chymotrypsin-like fold with cysteine as the active-site nucleophile, similarly to the 3C protease of picornaviruses and the main protease of coronavirus [2,3]. The protease has the essential role of cleaving the polyprotein precursor into the individual mature nonstructural proteins [4], besides hampering the cellular immune response by cleavage of the mitochondrial antiviral signaling protein (MAVS) [5]. Protease-targeting antivirals have been developed to treat infections with human immunodeficiency virus (HIV), hepatitis C virus (HCV) or studied in clinical trials for human rhinovirus (HRV) [6]. Rupintrivir (AG 7088), a protease inhibitor that was originally designed to treat infections with HRVs, also showed antiviral activity against other viruses such as picorna-, corona- and caliciviruses but was not further developed [7,8,9,10,11].

## 2. Materials and Methods

### 2.1. Viruses and Cells

Murine norovirus (MNV, strain MNV 1.CW1) was propagated and RAW 264.7 cells were maintained as described earlier [12]. HGT cells bearing the HuNoV GI.1 replicon were provided by Ian Goodfellow (University of Cambridge) and were maintained as described earlier [13]. Cells were incubated at 37 °C in a humidified atmosphere of 5% CO_2_. To obtain the MNV virus stock, once full cytopathic effect (CPE) was observed, cells underwent two freeze–thaw cycles, and the virus was harvested from the supernatant after centrifugation (10 min, 1000× *g*) and stored at −80 °C. The viral titer was determined by endpoint titration.

### 2.2. Compounds

**Scheme 1:**
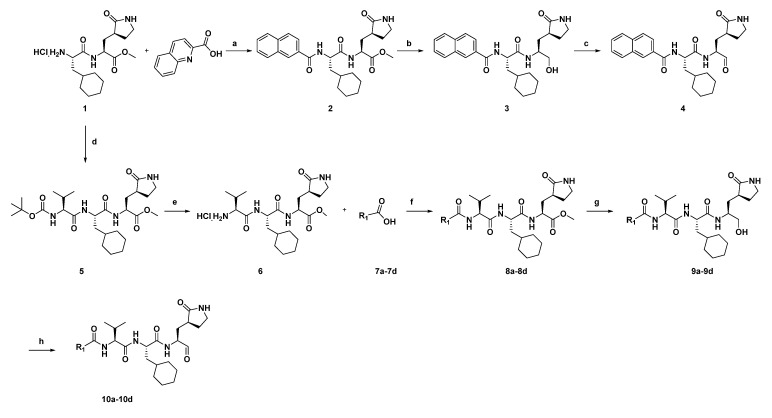
Reagents and conditions. (**a**) HATU, DIPEA, CH_2_Cl_2_, 20 °C, 12 h; (**b**) NaBH_4_, CH_3_OH, CH_2_Cl_2_; (**c**) Dess–Martin periodinane, CH_2_Cl_2_; (**d**) *N*-(tert-Butoxycarbonyl)-*L*-valine, HATU, DIPEA, CH_2_Cl_2_, 20 °C, 12 h; (**e**) 4 M HCl, 12 h; (**f**) HATU, DIPEA, CH_2_Cl_2_, 20 °C, 12 h; (**g**) NaBH_4_, CH_3_OH, CH_2_Cl_2_; (**h**) Dess–Martin periodinane, CH_2_Cl_2_.

Compounds were designed and synthesized as showed in Scheme 1 and in the Supplementary Materials and Methods. The materials and solvents were purchased from commercial sources and used without further purification. All products were characterized by the NMR and MS spectra; 1H and 13C NMR spectra were recorded on a 400 MHz, 500 MHz or 600 MHz instrument. Compounds were purified by chromatography with silica gel (300–400 mesh). Analytical thin-layer chromatography (TLC) was performed on HSGF 254 (0.15–0.2 mm thickness) sheets, and preparative thin-layer chromatography (PTLC) was performed on HSGF 254 (0.4–0.5 mm thickness) sheets. High-resolution mass spectra (HRMS) were measured on a Micromass Ultra Q-Tof spectrometer. For the antiviral experiments, compounds were dissolved in dimethyl sulfoxide (DMSO, VWR Chemicals, Leuven, Belgium).

The synthetic route of the compounds (**4** and **10a**–**10d**) is shown in Scheme 1, and the starting material for compound **1** was synthesized as described earlier [14]. The ester of compound **2** was condensed from compound **1** with **2**-quinolinecarboxylic acid, and then the intermediate of compound **2** was subsequently reduced by NaBH_4_ to generate the corresponding alcohol (compound **3**) which was oxidized into aldehydes (compound **4**) with Dess–Martin periodinane (DMP). The other compounds (**10a**–**10d**) were also synthesized from the starting material of compound **1**, and the intermediate (compound **5**) was obtained from compound **1** and a *N*-(tert-Butoxycarbonyl)-*L*-valine. Subsequently, the intermediate (compound **6**) was obtained by removing the *t*-butoxycarbonyl group of compound **5**. Coupling compound **6** with the corresponding acid (compounds **7a**–**7d**) yielded the esters intermediate (compounds **8a**–**8d**). Then the peptidomimetic aldehydes (compounds **10a**–**10d**) were generated from compound **8a**–**8d** via a two-step route. The synthesis procedure of the compounds can be found in the Supplementary Materials data.

### 2.3. Antiviral and Cytotoxicity Assay

Murine norovirus: The experiment was performed as described earlier [12]. In short, RAW 264.7 cells (1 × 10^4^ cells/well) were infected with MNV.CW1 in the presence of a dilution series of compounds. Antiviral activity and cytotoxicity were determined using the MTS method. The 50% effective concentration (EC_50_) was defined as the compound concentration that protected 50% of the cells from CPE. The % cell viability was calculated as (OD_treated_/OD_CC_) × 10, and the 50% cytotoxicity concentration (CC_50_) was defined as the compound concentration that reduces the number of viable cells by 50%.

HuNoV GI.1 replicon: The experiment was performed as described earlier with minor modifications [15]. In short, HGT cells (3 × 10^2^ cells/well) were seeded into the wells of a 96-well plate without G418. After 24 h of incubation, serial dilutions of compounds were added. Cells were further incubated for 72 h, and then cells were collected for RNA load quantification by RT-qPCR. To determine relative levels of Norwalk virus replicon RNA, β actin was used as a normalizer and ratios were calculated as previously described [15]. The EC_50_ values were defined as the compound concentration that resulted in 50% reduction of the relative HuNoV GI.1 replicon RNA levels.

### 2.4. Virus Yield Assay and RT-qPCR

The supernatants of treated or untreated infected cells were harvested. The viral RNA was isolated from supernatant using the NucleoSpin RNA Virus Kit (MACHEREY-NAGEL, Düren, Germany), according to the manufacturer’s protocol, and was quantified by a one-step RT-qPCR (Bio-Rad, Hercules, CA, USA). The primers, probe and cycling conditions were described earlier [16,17].

### 2.5. Generation of Drug-Resistant MNV Variants

RAW 264.7 cells (1 × 10^4^ cells/well) were seeded into the wells of 96-well plates and were infected with MNV.CW1 in the presence of a dilution series of compound **4**. When CPE was visible in the virus control (no compound), all the wells were scored for visible CPE. Virus was harvested from the wells in which CPE was observed under the highest compound pressure. The resistant virus was then diluted 1:5 and used to infect a new 96-well plate under the same conditions to continue. After 30 passages (~7 months), the viral RNA was isolated using the NucleoSpin RNA Virus Kit (MACHEREY-NAGEL Düren, Germany), after which a one-step RT-PCR (QIAGEN, Hilden, Germany) was performed and the sample sent for sequencing. Primers were 2950(+): 5′ accgccaggtcgactacg 3′, 3981(-): 5′ tcatacatgttgttggcgtg 3′. Sequences were analyzed and mutations were detected using the Geneious software version 2021.1.1 (Auckland, New Zealand). An untreated virus control was passaged in parallel and sequenced at the same time point to determine the background mutations occurring during serial passaging.

### 2.6. HuNoV GI.1 Replicon Clearance Assay

The experiment was performed as described earlier with minor modifications [11]. In short, HuNoV GI.1 replicons bearing HGT cells were seeded (2.5 × 10^5^ cells) in 25 cm^2^ T-flasks, with G418. The next day, cell culture medium was replaced for DMEM without G418 containing 10 μM of compound **4** or no compound. When 90% confluent (~3 days later), cells were trypsinized: (i) 2.5 × 10^5^ cells were seeded in a new 25 cm^2^ T-flask with the same concentration of compound **4** or no compound, (ii) 2.5 × 10^5^ cells were seeded in a new 25 cm^2^ T-flask without compound but with 1.25 mg/mL compound **4** (rebound step), and (iii) 1.5 × 10^5^ cells from each flask were collected for quantification of replicon/β actin RNA.

### 2.7. Generation of Drug-Resistant HuNoV GI.1 Replicon Variants

HuNoV GI.1 replicons bearing HGT cells were seeded (2.5 × 10^5^ cells) in 25 cm^2^ T-flasks, with G418 and 1 µM of compound **4** (EC_50_ value). When 90% confluent (after ~3 days), cells were trypsinized: (i) 2.5 × 10^5^ cells were seeded in a new 25 cm^2^ T-flask with the same concentration of compound **4**, and (ii) 1.5 × 10^5^ cells were collected and stored at −80 °C for isolation of viral RNA using the RNeasy Mini Kit (QIAGEN, Hilden, Germany). This was repeated once more before the concentration of compound **4** was increased 2-fold. A one-step RT-PCR (QIAGEN, Hilden, Germany) was performed with HuNoV GI.1 primers (FW: 5′–gccgtggtctgagtgatg–3′, REV: 5′–ccaccatcaccacctgc–3′, 5′–caccatcaccacctgc–3′) and sent for Sanger sequencing (Macrogen Inc). Sequences were analyzed and mutations were detected using the Geneious software version 2021.1.1 (Auckland, New Zealand). Untreated HGT cells were passaged in parallel and sequenced at the same time point to determine the background mutations occurring during serial passaging.

### 2.8. Cell-Based Fluorescence Resonance Energy Transfer (FRET) Assay

The assay was performed as described previously [18,19]. In short, HEK293T cells (5 × 10^4^ cells/well) were seeded into black 96-well cell plates (Greiner, Vilvoorde, Belgium) in the presence of a dilution series of compounds, and cells were incubated at 37 °C in a humidified atmosphere of 5% CO_2_. After 4 h, the cells were transfected with 0.1 μg of protease plasmid and 0.1 μg of the FRET plasmid (kindly provided by Prof. I. Goodfellow, University of Cambridge) with the TransIT^®^ LT1 Transfection Reagent (Mirus Bio LCC, Madison, WI, USA) according to the manufacturer’s protocol. The following day, the medium was removed and replaced with PBS (Gibco, Waltham, MA, USA). The fluorescent levels were analyzed on a Spark plate reader (Tecan, Männedorf, Switzerland), with excitation set at 434 nm and the reading emissions set at 477 nm cyan fluorescent protein (CFP) and 527 nm yellow fluorescent protein (YFP). The background fluorescence was measured and subtracted from wells transfected with an empty vector (pUC18). The 50% inhibitory concentration (IC_50_) values were calculated with nonlinear fitting in GraphPad version prism 7 (San Diego, CA, USA).

### 2.9. Western Blot

To detect the cleavage of the FRET sensor and the presence of the NS6 viral protein activity, HEK293T cells were harvested 24 h post transfection for western blot analysis. Transfected cells were lysed in RIPA buffer (Thermo Scientific, Waltham, MA, USA) in the presence of a protease inhibitor (Halt Protease Inhibitor Cocktail kit), centrifuged at 9000 g for 5 min at 4 °C and sonicated for 4 times 5 min at 4 °C. Protein concentration of the lysates was determined using the BCA protein assay kit (Thermo Scientific, Waltham, MA, USA). Three µg of cell lysates were analyzed by SDS-PAGE and Western blotting. Proteins were separated by SDS-PAGE (Criterion XT gel, 10% Bis-Tris, Bio-Rad, Hercules, CA, USA) and transferred onto a PVDF membrane (Trans-Blot Turbo, Bio-Rad, Hercules, CA, USA). After blocking with 1% PBS T milk for 1 h, the membranes were incubated overnight with an anti-RFP antibody (ChromoTek, 6G6), anti-GFP antibody (ChromoTek, PABG1, Planegg, Germany) or anti-GAPDH antibody (Sigma, SAB2701826, Darmstadt, Germany) at a 1:1000 dilution. The next day, the membranes were washed extensively and incubated with species-specific horseradish peroxidase (HRP) conjugated secondary antibodies (Dako). Proteins were detected using chemiluminescence according to the manufacturer’s instructions (Thermo Scientific, SuperSignal West Femto Maximum Sensitivity Substrate, Waltham, MA, USA) using the ChemiDoc MP Imaging System.

### 2.10. Site-Directed Mutagenesis

The desired mutations were introduced into the protease cDNA clones using the QuikChange II XL mutagenesis kit (Agilent, Santa Clara, CA, USA) according to the manufacturer’s instructions. The presence of the desired mutation in the produced construct was confirmed by PCR, and Sanger sequencing was performed (Macrogen Inc., Seoul, Korea).

### 2.11. Antiviral Treatment of HuNoV-Infected Zebrafish Larvae via Injections in the Pericardial Cavity

Zebrafish larvae were anesthetized and positioned as previously [20,21], in short: zebrafish larvae of three days post fertilization (dpf) were anesthetized and transferred to an agarose mold to position them on their dorsal side with the yolk facing upward. In every experiment, the injection needle was calibrated to ensure the precision of the injection volume. Microinjection was performed using an M3301R Manual Micromanipulator (WPI, Friedberg, Germany) and a FemtoJet 4i pressure microinjector (Eppendorf, Hamburg, Germany). Compound-treated zebrafish larvae received a 1 nL injection of compound in pericardial cavity, while negative control zebrafish were injected with 1 nL of phosphate-buffered saline (PBS). Zebrafish larvae were injected in the yolk with 3 nL of virus or with 3 nL of PBS (negative control).

After injection, zebrafish larvae were transferred to 6-well plates with Danieau’s solution and further maintained in an incubator with a 14/10 h light/dark cycle at 32 °C. Every day post injection, the general condition of the zebrafish larvae (e.g., posture, swimming behavior or signs of edema) was observed in order to record clinical signs of virus infection, and 10 zebrafish larvae were collected and stored at 80 °C.

### 2.12. Tissue Homogenization, RNA Extraction and RT-qPCR-qPCR for Detection of Human Norovirus

Zebrafish larvae were homogenized (Precellys 24, Bertin Technologies, Montigny-le-Bretonneux, France), the homogenates were cleared by centrifugation (5 min, 9000× *g*), and RNA was extracted using the RNeasy Mini Kit (QIAGEN, Hilden, Germany), according to the manufacturer’s protocol. RNA levels were quantified with a one-step RT-qPCR (iTaq Universal Probes One-Step Kit, Bio-Rad, Hercules, CA, USA) as previously described [20,21].

### 2.13. Pharmacokinetic Studies in Mice

Male CD-1 mice (3 per group, weight 18–28 g) were treated with a solution of compound **4** or **10d** (DMSO/EtOH/PEG300/NaCl (5/5/40/50, *v*/*v*/*v*/*v*)) at doses of 20 mg/kg via intraperitoneal administration, 5 mg/kg via subcutaneous administration or 5 mg/kg via intraperitoneal administration. Blood samples were collected at 0.05, 0.25, 0.75, 2, 4, 8 and 24 h after administration. Serum samples were obtained through common procedures, and the concentrations of compound in the supernatant were analyzed by LC-MS/MS. All procedures relating to animal handling, care and treatment were performed according to the guidelines approved by the Institute Animal Care and Use Committee at Shanghai Institute of Materia Medica.

## 3. Results

**Figure 1:**
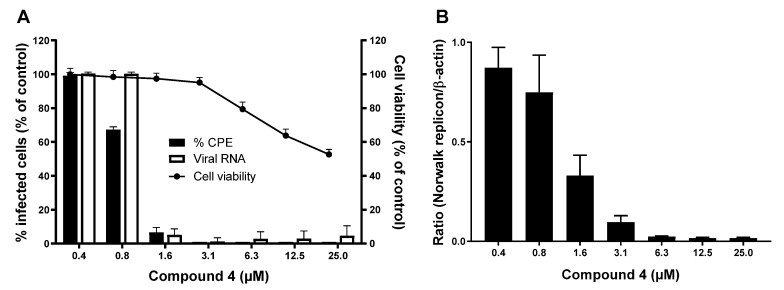
The antiviral effect of compound **4** against MNV and the HuNoV GI.1 replicon. (**A**) The antiviral activity of compound **4** against MNV was quantified by (i) virus-induced CPE reduction and (ii) reduction in viral RNA levels. Cellular toxicity was assessed in parallel. *n* = 3–4 (±SEM). (**B**) The antiviral activity of compound **4** against the HuNoV GI.1 replicon. Intracellular RNA loads are represented by relative genome quantification of the HuNoV GI.1 replicon versus β-actin. *n* = 5 (±SEM).

In the search for a norovirus-protease-targeting antiviral, we screened ~300 peptidomimetic aldehydes. Compound **4** (Figure 1) inhibited mouse norovirus (MNV)-induced CPE and viral RNA with an EC_50_ value of 0.7 ± 0.2 µM and 1.7 ± 0.6 µM, respectively. A CC_50_ value of 25.3 ± 6.1 µM was determined, resulting in a selectivity index (SI) of 35.6. In addition, compound **4** also inhibited the HuNoV GI.1 replicon (EC_50_: 1.2 ± 0.6 µM) and showed no adverse effects on the host cells up to the hi0 ghest tested concentration of 100 µM.

**Figure 2:**
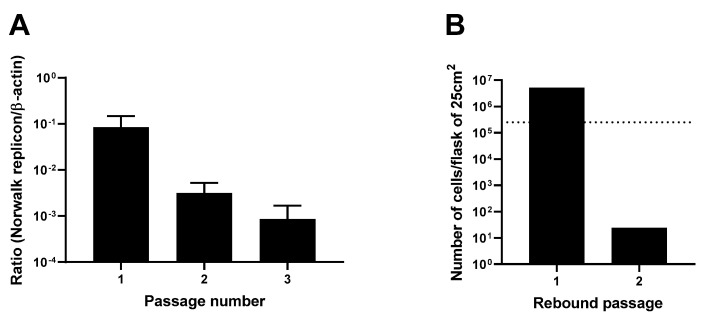
Clearance of the HuNoV GI.1 replicon from HGT cells by compound **4.** (**A**) Clearance phase: cells were treated for three consecutive passages with compound **4** (10 µM) without G418. (**B**) Rebound phase: cells from the clearance phase were placed under selective pressure with G418, without compound **4**. The dotted line represents the minimum number of HGT cells required for the next passage.

Next, HuNoV GI.1 replicon bearing HGT cells were treated with 10 µM (~10x EC_50_) in multiple passages [15]. This resulted in a 2 log_10_ reduction in HuNoV GI.1 RNA (Figure 2A) after two passages and in a 3 log_10_ reduction after three passages. Compound **4** could clear cells from the HuNoV GI.1 replicon completely, as after two consecutive treatments with 10 µM, the HGT cells had lost the ability to replicate with G418 (Figure 2B).

To evaluate the dynamics of emerging resistance against compound **4**, cells containing MNV and HuNoV GI.1 replicon were cultured under sub-optimal concentrations. After 25 passages (6 months), a weak compound **4**^res^ MNV variant developed. The MNV compound **4**^res^ variant had a minor shift in EC_50_ of 4.5-fold. However, no mutations in the protease gene were found that could be linked to the resistant phenotype. Similarly, HGT cells carrying the HuNoV GI.1 replicon were cultured under sub-optimal concentrations. After three months, a compound **4**^res^ HuNoV GI.1 replicon variant was selected, with a shift in EC_50_ of 13.8-fold (Table 1). Similarly, we selected for a rupintrivir-resistant HuNoV GI.1 replicon variant, with the compound pressure up to 14 µM, and a shift in EC_50_ of 15-fold was obtained (Table 1).

When compound **4**^res^ HGT cells were treated with rupintrivir, a shift in EC_50_ of 10.8-fold was observed (EC_50_ 10.9 ± 4.17 µM). While treatment of rupintrivir^res^ HGT cells with compound **4** resulted in a smaller shift of 6.8-fold (EC_50_ 5.67 ± 0.73 µM). Sequence analysis of the compound **4**^res^ HuNoV GI.1 replicon variant revealed one mutation in the protease (I109V); the selected rupintrivir^res^ variant carried another mutation (A105V). These amino acids are highly conserved within the *Caliciviridae* (Figure S1). The I109V mutation was previously associated with in vitro resistance to rupintrivir; a double A105V/I109V mutation was also reported [22].

**Figure 3:**
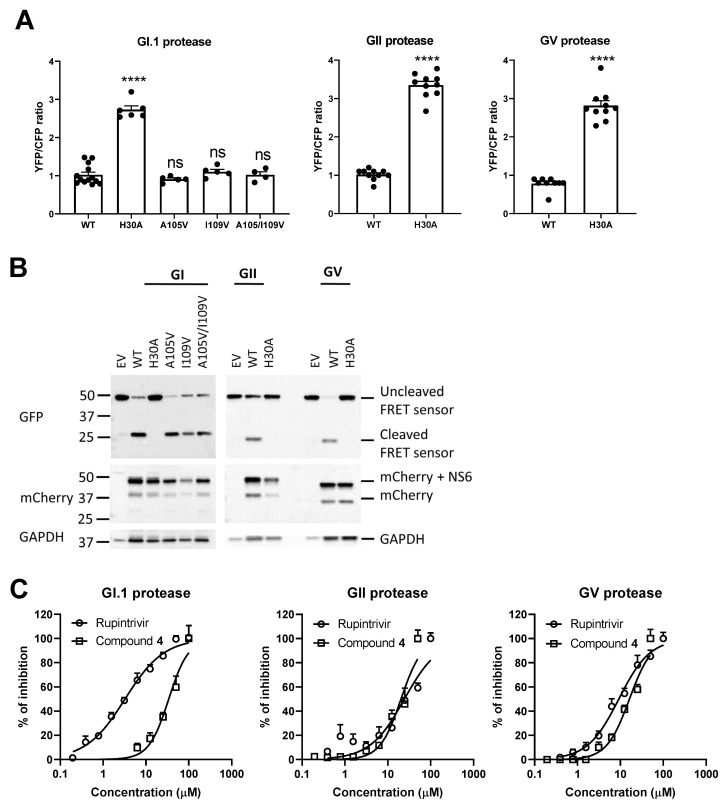
Compound **4** and rupintrivir block the norovirus protease. (**A**) The FRET signal was lost upon co-transfection with the wild-type (WT) or a mutated protease (A105V, I109V, A105V/I109V). The signal remained upon co-transfection with the inactive (H30A) protease. (**B**) Western blot of HEK293T cells co-transfected with the GI, GII or GV WT or mutant proteases along with the FRET construct. Cleavage was observed in the WT and mutant proteases in all genogroups. EV: empty vector. (**C**) Dose–response curves of compound **4** and rupintrivir on the WT proteases. Mean values ±SEM are presented, Mann–Whitney test **** *p* < 0.0001, ns: not significant.

To confirm that compound **4** acts by inhibiting the proteolytic activity of the HuNoV protease and the relevance of the identified resistant mutations, we studied the activity of the compound in a FRET-based protease assay [18]. When the protease is absent, blocked or inactive (H30A), a high YFP signal but a low CFP signal is detected (Figure 3A). In contrast, the wild-type (WT) or mutated proteases (A105V, I109V, A105V/I109V) show strong cleavage of the FRET linker, resulting in a low YFP signal but a high CFP signal. We confirmed the presence of the protease and that the loss of the FRET signal was linked to a successful cleavage (Figure 3B). A dose-dependent effect was noted when compound **4** or rupintrivir was added to cells transfected with one of the norovirus proteases (Figure 3C). Rupintrivir inhibited the protease with IC_50_ values of 3 µM (CI:2.5–3.5 µM) for the GI.1 protease, 12.2 µM (CI: 9.1–16.3 µM) for the GII protease and 4.6 µM (CI: 3.8–5.5 µM) for the GV protease; these values are comparable with a previous study [22]. Compound **4** was less potent, with IC_50_ values of 34 µM (CI: 28.6–40.31 µM) for the GI.1 protease, 40.6 µM (CI: 34.9–47.2 µM) for the GII protease and 32.5 µM (CI: 28.5–37.0 µM) for the GV protease.

**Figure 4:**
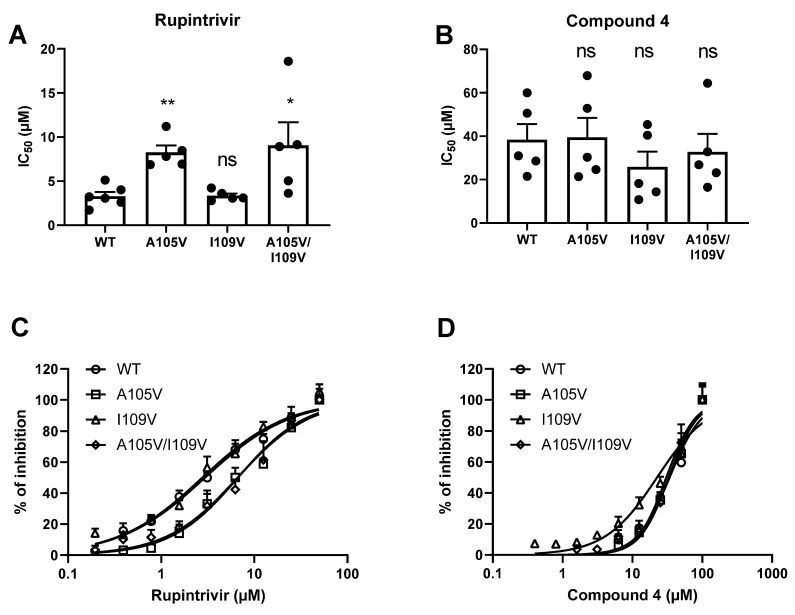
The susceptibility of mutated GI.1 proteases to rupintrivir and compound **4**. IC_50_ values of rupintrivir (**A**) and compound **4** (**B**) on the WT or mutant GI proteases. Dose–response inhibition curves of rupintrivir (**C**) and compound **4** (**D**) on the WT or mutant GI proteases. Mean values ±SEM are presented, Mann–Whitney test ** *p* < 0.01, * *p* < 0.05, ns: not significant.

We confirmed that the mutant GI.1 proteases still had a strong cleavage activity, comparable to that of the WT protease (Figure 3A,B). In the FRET assay, the A105V mutation and the A105V/I109V mutation in a GI.1 protease resulted in a significant shift in IC_50_ value for rupintrivir but not for compound **4**. The single I109V mutation did not result in a significant reduction of the GI.1 protease sensitivity to rupintrivir or compound **4** (Figure 4).

Molecular-docking studies indicate that compound **4** can occupy the 3CL protease (GI.1 and GII.4 norovirus) active site (Figure S2). The aldehyde group of compound **4** can form a C-S covalent bond with 3CL protease, and the (S)-lactam ring occupies the S1 subsite of the 3CLprotease. In GI.1 norovirus 3CL^pro-^ compound **4**, the S2 subsite can be occupied by the cyclohexyl, while the cyclohexyl cannot fit the S2 subsite of GII.4 norovirus 3CL^pro^.

As the initial hit, compound **4**, did not show a superior antiviral effect to rupintrivir, novel derivatives were synthetized to increase the potency of this class (Table 2).

**Figure 5:**
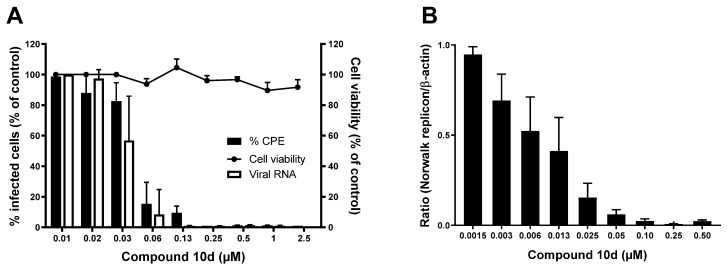
The antiviral effect of compound **10d** against MNV and the HuNoV GI.1 replicon. (**A**) The antiviral activity of compound **10d** against MNV was quantified by (i) virus-induced CPE reduction and (ii) reduction in viral RNA levels. Cellular toxicity was assessed in parallel. *n* = 3–4 (±SEM). (**B**) The antiviral activity of compound **10d** against the HuNoV GI.1 replicon. Intracellular RNA loads are represented by relative genome quantification of the HuNoV GI.1 replicon versus β-actin. *n* = 3 (±SEM).

Of the active analogues, compound **10d** (Figure 5) showed to have a very potent inhibitory effect against both GI and GV noroviruses. It inhibited MNV-induced CPE with an EC_50_ value of 0.037 ± 0.016 µM and viral RNA synthesis with an EC_50_ value of 0.03 ± 0.01 µM, and a CC_50_ value of 48 ± 3 µM was determined, resulting in a selectivity index (SI) of >1000. In addition, compound **10d** showed strong antiviral activity against the HuNoV GI.1 replicon with an EC_50_ value of 0.012 ± 0.010 µM and without adverse effects on the host cells up to the highest tested concentration of 100 µM.

**Figure 6:**
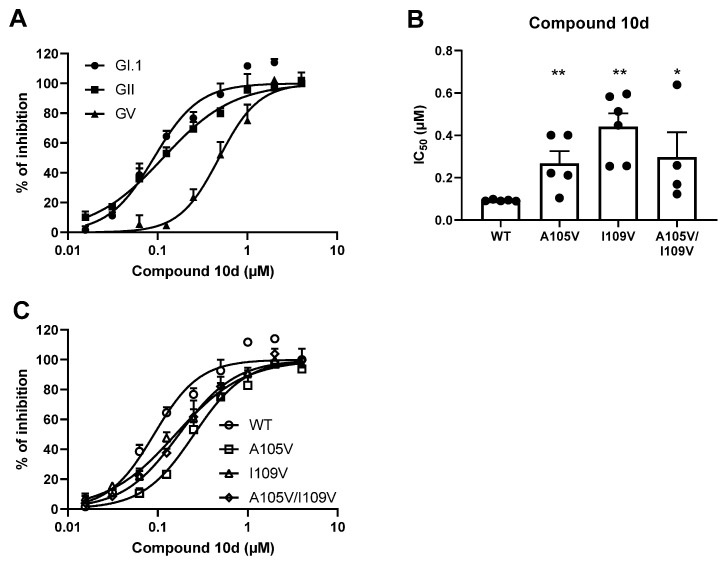
The susceptibility of mutated GI.1 proteases to compound **10d**. (**A**) Dose–response inhibition curves showing the effect of compound **10d** on the WT proteases. (**B**) IC_50_ values of compound **10d** on the WT or mutant GI.1 proteases. (**C**) Dose–response inhibition curves showing the effect of compound **10d** on the WT or mutant GI proteases. Mean values ±SEM are presented, Mann–Whitney test, ** *p* < 0.01, * *p* < 0.05.

Also in the FRET protease assay, compound **10d** was much more potent in blocking the different proteases than compound **4** and rupintrivir (Figure 6A). Treatment with compound **10d** resulted in IC_50_ values of 0.09 µM (CI: 0.08–0.11 µM) for the GI.1 protease, 0.12 µM (CI: 0.09–0.14 µM) for the GII protease and 0.49 µM (CI: 0.42–0.56 µM) for the GV protease. Likewise, we investigated if the mutations (A105V, I109V, A105V/I109V) affected the susceptibility of the HuNoV GI.1 protease to compound **10d**. The GI.1 protease was less susceptible to the compound in the presence of each of the mutations (Figure 6B). The I109V mutation yielded a greater shift in IC_50_ value (2.5-fold), while the A105V and A105V/I109V resulted in a 1.6-fold change (Figure 6C).

**Figure 7:**
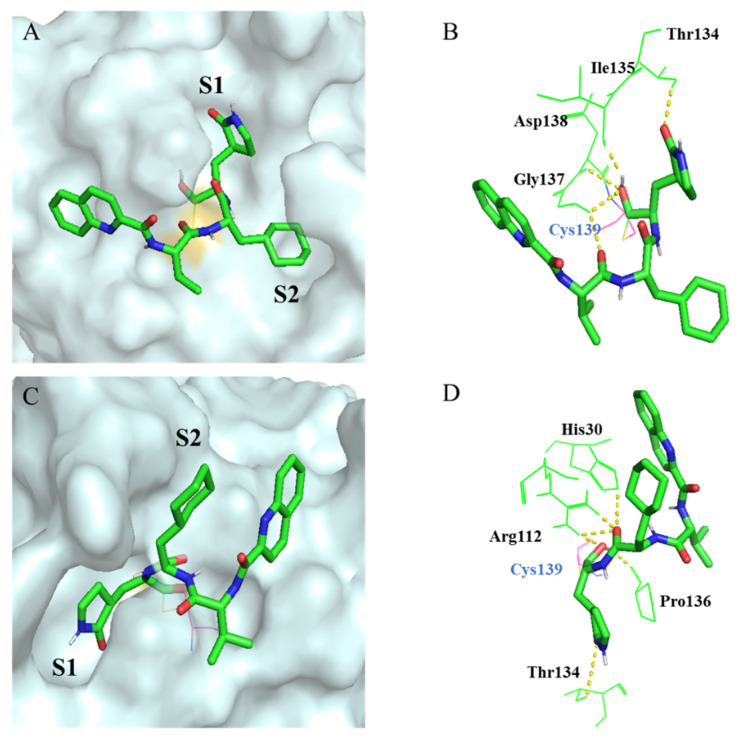
Molecular docking of compound **10d** in the GI.1 and GII.4 norovirus 3CL protease. (**A**) The binding mode of **10d** at the substrate-binding site of the GI.1 norovirus 3CL^pro^ (PDB code: 3UR9). The GI.1 norovirus 3CL^pro^ was shown as molecular surface and **10d** was shown by green sticks. (**B**) Interactions of **10d** with the surrounding residues. Residues are shown as light green lines, and H-bonds are represented by yellow dashed lines. (**C**) The binding mode of **10d** at the substrate-binding site of the GII.4 norovirus 3CL^pro^ (PDB code: 6NIR). (**D**) Interactions of **10d** with the surrounding residues. The Schrödinger program (Maestro covalent docking) was used for calculations and PyMOL program for visualizations.

Molecular docking indicates that compound **10d** occupies the 3CL protease (GI.1 norovirus) and GII.4 norovirus active site well (Figure 7). The interacted model of GI.1 norovirus 3CL^pro^ compound **10d** shows that the aldehyde group of compound **10d** plays a crucial role in maintaining the antiviral activities. The carbon atom of the aldehyde group can bind with Cys139, and the oxygen atom forms a hydrogen bond with Ile135, Gly137 and Asp138. The (*S*)-lactam ring occupies the S1 subsite, and the lactam group interacts with Thr134; furthermore, the cyclohexyl fits well into the S2 subsite. In addition, the amide bonds of compound **10d** can form hydrogen bonds with Gly137. The interacted model (Figure 7C,D) also shows that the compound **10d** can occupy the GII.4 norovirus 3CL^pro^ well as the (*S*)-lactam ring fits the S1 subsite and the cyclohexyl can occupy the S2 subsite.

The aldehyde group of compound **10d** also plays an important role against the GII.4 norovirus 3CL^pro^. The carbon atom of the aldehyde group can bind with Cys139, and the oxygen atom forms a hydrogen bond with Arg112 and Pro136. In addition, the (*S*)-lactam ring interacts with Thr134 in S1 subsite. The amine bonds of compound **10d** can also interact with His30 and Arg112. This explains why compound **10d** shows potent antiviral activity against GI.1 and GII.4 norovirus.

**Figure 8:**
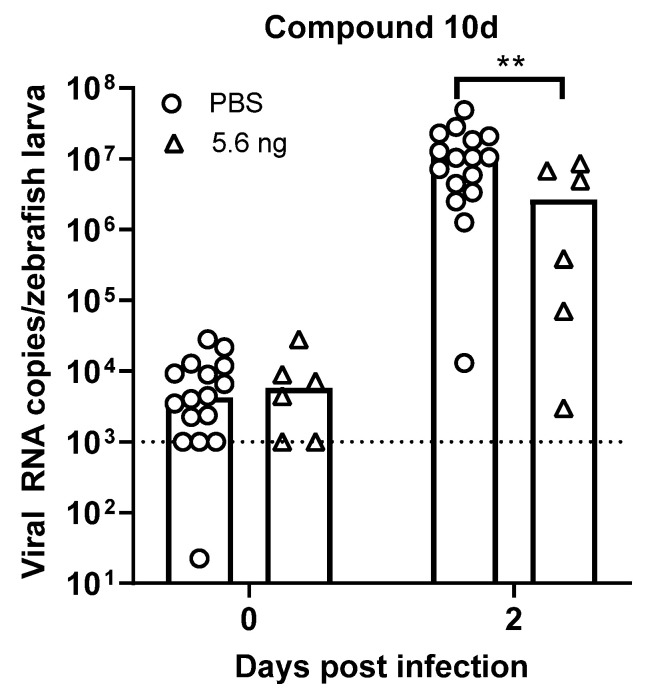
Compound **10d** reduces HuNoV GII.4 replication in zebrafish larvae. Single treatment of zebrafish infected with HuNoV GII.P4-GII.4 with compound **10d** (*n* = 6–11). The dotted line represents the limit of detection (LOD), mean values ±SEM, Mann–Whitney test, ** *p* < 0.01.

Finally, we evaluated whether these compounds have an in vivo antiviral effect against HuNoV in zebrafish larvae [20]. We injected 5.6 ng of compound **10d** in the pericardial cavity immediately before virus inoculation; adding the compound to the swimming water was not possible given the low water solubility. No toxic effects (abnormal body shape, impaired circulation/arrhythmia, impaired motility, edema) were observed with any of the concentrations tested. A single injection with 5.6 ng of compound **10** resulted in a 0.6 log_10_ reduction in HuNoV GII.4 viral RNA titers (*p* = 0.0062) at day 2 post infection, i.e., the peak of replication (Figure 8).

The pharmacokinetic properties of compounds **4** and **10d** were evaluated in CD-1 mice (Table 3). Compound **4** displayed a moderate half-life (T_1/2_), high maximal concentration, high area under the curve (AUC) value and good bioavailability. Compound **10d** displayed a long T_1/2_, high C_max_ value, AUC value and good bioavailability.

## 4. Discussion

In the search for an antiviral against HuNoV infections, we evaluated a compound library of ~300 compounds structurally related to the protease inhibitor rupintrivir for their potential anti-norovirus activity.

The early hit compound, compound **4**, showed to have an equipotent effect against GI and GV noroviruses. The activity of compound **4** against the HuNoV GI.1 replicon is comparable to rupintrivir; moreover, compound **4** could clear cells from the HuNoV GI.1 replicon thus confirming it has a strong antiviral effect. In addition, compound **4** performs better against MNV than rupintrivir (EC_50_: 13 ± 2 µM) [11]. We thus considered this as a promising class of compounds, given that protease inhibitors described thus far tend to be less potent against non-GI noroviruses when compared to GI [23].

The first attempt to obtain better insight into the mechanism of action was made by selecting for drug-resistant variants. We succeeded in selecting for a compound **4**^res^ HuNoV GI.1 replicon variant. Upon sequencing, a single mutation was detected in the protease (I109V), which had been previously associated with a weak resistance to rupintrivir [22]. Interestingly, when we selected for a rupintrivir^res^ HuNoV GI.1 replicon variant, another mutation was identified (A105V). Since we selected for rupintrivir-resistant clones that contain solely the A105V mutation, our data suggest that this could be more than a compensatory mutation, as was suggested by Kitano et al. The double mutation (A105V/A109V) that was reported before [22] was not found it in this study, perhaps due to differences in the protocol. Overall, the close position of the identified mutations and the (at least partial) cross-resistance observed lead us to hypothesize that compound **4** most likely interacts with the same residues of the HuNoV GI.1 protease and acts by (nearly) the same molecular mechanism as rupintrivir.

In the FRET assay, compound **4** was active against all proteases but 3- to 10-fold less potent than rupintrivir. This could be explained by the fact that this FRET assay might not take place in the same subcellular localization in which proteolytic cleavage takes place after a natural infection. Upon introduction of the mutations in the GI protease, we confirmed that the A105V and A105V/I109V but not the I109V mutation contributed to the reduced susceptibility to rupintrivir but not to compound **4**. This could be due to the rather low potency of the molecule in this assay, which does not help to make evident small shifts in IC_50_ values. In addition, the proteases containing the double mutant were not more resistant to rupintrivir than the single mutants. Taken together, one can conclude that additional mutations are necessary to result in full resistance and thus that the barrier to resistance of antivirals targeting the norovirus protease is high. This is an important plus point in favor of the development of such a class of antivirals against norovirus.

Aiming to improve the activity, additional derivatives were synthetized and tested. Of those, compound **10d** was about 100-fold more potent with EC_50_ values in the double-digit nanomolar range. This was further confirmed in the FRET assay, with a ~300-fold improvement against the GI and GII proteases. By using the mutated GI proteases, we found reduced susceptibility to compound **10d**, although this was a very low-grade resistance. This compound class shows to be less potent against GII than GI noroviruses, as other protease-targeting compounds [23]. Nevertheless, the best compound still has submicromolar potency against the GII protease (IC_50_ of 0.12 µM). It would be of interest to study the antiviral effect of this compound in human gut enteroids infected with GII HuNoV [24].

Finally, we evaluated the in vivo antiviral effect of compound 10d against HuNoV GII.4 [20]. Upon a single-treatment of compound **10d** immediately before virus inoculation, a 0.6 log_10_ reduction in viral RNA was observed at the peak of replication. This was achieved by a dose of around 22.5 ng/mg which is still a relatively low dose compared to rodents. Moreover, compounds **4** and **10d** have good PK properties in mice that warrant further development.

As the proteases of corona-, picorna- and caliciviruses have well-known structural similarities, this class of compounds was also tested against SARS-CoV-2 [14]. Hence, the further development of these compounds to treat COVID-19 patients could not only be an important breakthrough for the control of the current pandemic but also lead to the first approved and urgently needed antiviral treatment for norovirus infections. Of note is that protease inhibitors are known to have adverse effects [25,26], thus this aspect should be carefully assessed during their preclinical development. However, the current inhibitors targeting HIV and HCV protease are safe, and this class of compounds has thus far shown no reasons for concern regarding acute toxicity [14].

## 5. Conclusions

In conclusion, we described here a novel class of peptidomimetic aldehydes with potent antiviral activity against noroviruses in vitro and in a small animal model. Our findings highlight once more that the HuNoV protease is an attractive target for the development of norovirus antivirals with a high barrier to resistance. For the first time, a class of protease inhibitors was shown to be active against HuNoV GII.4 in a small animal model. Overall, we believe that these compounds should be further developed for the treatment of HuNoV-induced disease (as well as against corona- and enterovirus infections).

## Figures and Tables

**Table 1:** Selection of compound 4 and rupintrivir-resistant HuNoV GI.1 replicon variants.

Compound 4		
Selection Pressure	EC_50_ (µM)	Fold Change in EC_50_
DMSO control	0.8 ± 0.3	-
6 µM of compound **4**	3.8	4.5
10 µM of compound **4**	4.3	5.2
16 µM of compound **4**	11.5 ± 0.9	13.8
**Rupintrivir**		
**Selection Pressure**	**EC_50_ (µM)**	**Fold Change in EC_50_**
DMSO control	1.0 ± 0.1	-
10 µM of rupintrivir	9.4 ± 0.5	9.3
14 µM of rupintrivir	15 ± 3	15.2

**Table 2:**
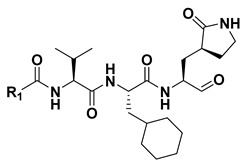
Structures of tetrapeptide inhibitors with R_1_ modifications and their antiviral activities against MNV and the HuNoV replicon.

No.	R_1_	MNV-EC_50_ (μM)	HuNoV-EC_50_ (μM)
**10a**	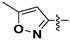	3.8 ± 0.97	0.24 ± 0.11
**10b**	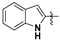	0.23 ± 0.11	0.016 ± 0.009
**10c**	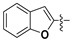	0.094 ± 0.014	0.016 ± 0.006
**10d**	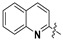	0.037 ± 0.016	0.012 ± 0.010

**Table 3:** Preliminary pharmacokinetic (PK) evaluation of compounds 4 and 10d.

Compd.	Route	T_1/2_	T_max_	C_max_	AUC_last_	AUC_INF_obs_	CL__obs_	MRT_INF_obs_	Vss__obs_	F
		(h)	(h)	(ng/mL)	(h·ng/mL)	(h·ng/mL)	(mL/min/kg)	(h)	(mL/kg)	(%)
**4**	i.p. (20 mg/kg)	5.11 ± 1.07	0.250	14,492 ± 2881	14,883 ± 1775	14,954 ± 1789	-	1.40 ± 0.06		72.1
	s.c. (5 mg/kg)	5.63 ± 0.40	0.58 ± 0.29	2196 ± 894	4057 ± 1142	4114 ± 1138	-	2.95 ± 0.53		78.6
	i.v. (5 mg/kg)	5.50 ± 0.85	-	-	5161 ± 1316	5193 ± 1302	16.8 ± 4.4	1.44 ± 0.30	1498 ± 714	
**10d**	i.p. (20 mg/kg)	4.26 ± 0.24	0.25	12,657 ± 5906	10,788 ± 3929	10,815 ± 3933	-	1.34 ± 0.29	-	150
	s.c. (5 mg/kg)	6.02 ± 0.92	0.42 ± 0.29	1568 ± 582	1843 ± 717	1857 ± 718	-	1.70 ± 0.16		102
	i.v. (5 mg/kg)	5.64 ± 1.63	-	-	1799 ± 63	1812 ± 67	46.0 ± 1.7	1.40 ± 0.23	3867 ± 758	

T_1/2_: half-life, i.p.: intraperitoneal administration, s.c.: subcutaneous administration, C_max_: maximal concentration, AUC: area under the curve, INF_obs: from time 0 extrapolated to infinite time, CL_obs_: clearance, MRT: mean residence time, Vss: steady-state volume of distribution, F: bioavailability.

## Data Availability

The data presented in this study are all presented in the manuscript.

## Supplementary Materials

The following are available online at https://www.mdpi.com/article/10.3390/v13091852/s1, Methods on synthesis procedure and compound data, Figure S1: Amino acid sequence alignment of norovirus proteases, Figure S2: Molecular docking was performed to study the potential interactions between compound **4** with the GI.1 and GII.4 norovirus 3CL^pro^.
