# Supplementary material for: A Novel Class of Norovirus Inhibitors Targeting the Viral Protease with Potent Antiviral Activity In Vitro and In Vivo
Source: Viruses. 2021 Sep 16;13(9):1852. doi: 10.3390/v13091852 (PMC8472913; doi:10.3390/v13091852)
Supplement: Supplementary file 1 [file viruses-13-01852-s001.zip › viruses-1359427-supplementary.pdf]

Supplementary Material

Synthesis procedure and compounds data.

Methyl (S)-2-((S)-3-cyclohexyl-2-(quinoline-2-carboxamido)propanamido)-3-((S)-2-oxopyrrolidin-3-yl)propanoate (2)

2-Quinolinecarboxylic acid (0.31 g, 1.8 mmol) was dissolved in 30 mL CH_2Cl_2 at -20 ℃, then the HATU (0.96 g, 2.5 mmol) was added. The solution was kept at -20 ℃ for 20 mins, and the intermediate 1 (0.68 g, 1.8 mmol) was added. After 30 mins later, the DIPEA (0.9 mL, 5.3 mmol) was added drop-wise, and the reaction mixture was stirred at -20 ℃ for 12 h. The resulting mixture was washed by 1 M HCl solution (60 mL×3), saturated NaHCO3 solution (60 mL×3) and brine (60 mL×3). The organic phase layer was dried over Na_2SO_4 and concentrated in vacuo. The resulting mixture residue was purified by column chromatography (DCM: CH_3OH, 40: 1 v/v) to afford the pure product 2 (0.74 g, 83 %) as a light solid.

^{1}H NMR (500 MHz, $CDCl_3$) δ 8.66 (d, J = 8.9 Hz, 1H), 8.28 (d, J = 8.5 Hz, 1H), 8.24 (d, J = 8.5 Hz, 1H), 8.14 (d, J = 8.6 Hz, 1H), 7.98 (d, J = 7.0 Hz, 1H), 7.85 (d, J = 7.7 Hz, 1H), 7.76 (ddd, J = 8.4, 6.9, 1.4 Hz, 1H), 7.61 (ddd, J = 8.1, 7.0, 1.1 Hz, 1H), 6.38 (s, 1H), 4.90 (td, J = 8.9, 5.9 Hz, 1H), 4.52 (ddd, J = 11.1, 7.1, 3.7 Hz, 1H), 3.74 (s, 3H), 3.30 (t, J = 9.0 Hz, 1H), 3.25 (td, J = 9.4, 6.8 Hz, 1H), 2.47-2.32 (m, 2H), 2.25 (m, 1H), 1.92-1.87 (m, 4H), 1.83-1.75 (m, 3H), 1.61 (d, J = 12.1 Hz, 1H), 1.52-1.45 (m, 1H), 1.22-0.92 (m, 6H).

^{13}C NMR (126 MHz, $CDCl_3$) δ 179.18, 172.25, 171.69, 163.91, 148.73, 146.09, 136.96, 129.66, 129.49, 128.88, 127.55, 127.16, 118.31, 51.96, 51.00, 50.64, 40.13, 39.96, 37.82, 33.57, 33.08, 32.48, 32.30, 29.22, 27.79, 25.97, 25.70.

LRMS (ESI) m/z: $[M+H]^+$ Found 495.0.

N-((S)-3-cyclohexyl-1-(((S)-1-hydroxy-3-((S)-2-oxopyrrolidin-3-yl)propan-2-yl)amino) -1-oxopropan-2-yl)quinoline-2-carboxamide (3)

The compound 2 (0.69 g, 1.4 mmol) was dissolved in dry THF (100 mL), and the NaBH4 (0.42 g, 11.2 mmol) was added slowly at 0 ^{o}C. Then the reaction mixture was stirred at room temperature for 3 h. The completion of the reaction was confirmed by TLC then the reaction was quenched and concentrated to get a crude residue. The residue was dissolved in DCM, and washed with saturated ammonium chloride solution (50 mL×3), saturated $NaHCO_3$ solution (50 mL×3) and brine (50 mL×3). The organic phase was dried over Na_2SO_4 and concentrated, and the residue was purified by column chromatography (DCM: CH_3OH, 20: 1 v/v) to afford the pure product 3 (0.55g, 85 %) as a light solid.

^{1}H NMR (500 MHz, $CDCl_3$) δ 8.65 (d, J = 8.5 Hz, 1H), 8.25 (d, J = 8.5 Hz, 1H), 8.20 (d, J = 8.5 Hz, 1H), 8.12 (d, J = 8.3 Hz, 1H), 7.83 (dd, J = 7.7, 3.9 Hz, 2H), 7.74 (ddd, J = 8.4, 6.9, 1.4 Hz, 1H), 7.59 (ddd, J = 8.1, 6.9, 1.1 Hz, 1H), 6.56 (s, 1H), 4.79 (td, J = 8.8, 5.8 Hz, 1H), 4.05 (m 1H), 3.95 (s, 1H), 3.65 (s, 2H), 3.24 (dd, J = 16.1, 8.4 Hz, 1H), 3.19 (dt, J = 16.5, 8.2 Hz, 1H), 2.44 (m, 1H), 2.31 (m, 1H), 2.05 (m, 1H), 1.85 (m, 2H), 1.81-1.69 (m, 3H), 1.68-1.61 (m, 2H), 1.60-1.54 (m, 2H), 1.41 (ddd, J = 11.1, 8.7, 4.1 Hz, 1H), 1.21-1.07 (m, 3H), 0.96 (m, 2H).

^{13}C NMR (126 MHz, $CDCl_3$) δ 180.53, 172.72, 164.08, 148.66, 146.04, 136.99, 129.67, 129.44, 128.86, 127.58, 127.16, 118.31, 65.39, 51.20, 50.05, 40.08, 37.70, 33.78, 33.11, 32.26, 31.66, 27.93, 25.91, 25.71, 25.58.

LRMS (ESI) m/z: $[M+H]^+$ Found 467.0.

N-((S)-3-cyclohexyl-1-oxo-1-(((S)-1-oxo-3-((S)-2-oxopyrrolidin-3-yl)propan-2-yl)amino)propan-2-yl)quinoline-2-carboxamide (4)

To a solution of the 3 (0.47 g, 1.0 mmol) in CH_2Cl_2, DMP (0.5 g, 1.2 mmol) was added slowly and the reaction mixture was stirred at room temperature for 5h. The completion of the reaction was confirmed by TLC then quench and concentrate the reaction, and the reaction was filtered and washed with saturated $NaHCO_3$ solution (50 mL×3) and brine (50 mL×3). The organic phase was dried over $MgSO_4$ and concentrated, and the residue was

purified by column chromatography (DCM: CH_3OH, 20: 1 v/v) to afford the pure product 1 (0.32 g, 70 %) as a light solid.

^1H NMR (600 MHz, Acetone-d_6) δ 9.58 (s, 1H), 8.84 (d, *J* = 8.4 Hz, 1H), 8.51-8.45 (m, 1H), 8.23 (d, *J* = 8.4 Hz, 1H), 8.12 (d, *J* = 8.6 Hz, 1H), 8.01 (t, *J* = 6.7 Hz, 1H), 7.85-7.79 (m, 1H), 7.70-7.63 (m, 1H), 7.20 (s, 1H), 4.96-4.84 (m, 1H), 4.41 (m, 1H), 3.25 (m, 2H), 3.15 (m, 1H), 2.59-2.39 (m, 1H), 2.35-2.26 (m, 1H), 2.06 (dd, *J* = 4.4, 2.1 Hz, 1H), 1.95-1.77 (m, 5H), 1.65 (dd, *J* = 7.9, 2.9 Hz, 2H), 1.59-1.52 (m, 2H), 1.30-1.04 (m, 4H), 1.02-0.90 (m, 2H).

^{13}C NMR (151 MHz, Acetone-d_6) *δ* 199.48, 178.93, 172.46, 163.45, 149.36, 146.02, 137.28, 129.89, 129.14, 127.65, 127.52, 118.21, 57.04, 50.77, 40.35, 39.61, 37.57, 33.73, 33.07, 32.10, 29.29, 27.61, 25.84, 25.62.

HRMS (ESI) m/z: [M+H]+ Calcd for C26H33N4O4: 465.2496; Found 465.2506.

Methyl (9S,12S)-9-(cyclohexylmethyl)-6-isopropyl-2,2-dimethyl-4,7,10-trioxo-12-(((S) -2-oxopyrrolidin-3-yl)methyl)-3-oxa-5,8,11-triazatridecan-13-oate (5)

Boc-*L*-Val-OH (0.39 g, 1.8 mmol) was dissolved in 30 mL CH_2Cl_2 at -20 °C, then the HATU (0.96 g, 2.5 mmol) was added. The solution was kept at -20 °C for 20 mins, and the intermediate 1 (0.68 g, 1.8 mmol) was added. After 30 mins later, the DIPEA (0.9 mL, 5.3 mmol) was added drop-wise, and the reaction mixture was stirred at -20 °C for 12 h. The resulting mixture was washed by 1 M HCl solution (60 mL×3), saturated $NaHCO_3$ solution (60 mL×3) and brine (60 mL×3). The organic phase layer was dried over Na_2SO_4 and concentrated in vacuo. The resulting mixture residue was purified by column chromatography (DCM: CH_3OH, 40: 1 v/v) to afford the pure product 4 (0.68 g, 70 %) as a light solid

^1H NMR (500 MHz, $CDCl_3$) *δ* 7.86 (d, *J* = 7.2 Hz, 1H), 7.41 (d, *J* = 7.4 Hz, 1H), 7.20 (s, 1H), 5.26 (d, *J* = 8.5 Hz, 1H), 4.63 (m, 1H), 4.51 (s, 1H), 3.87 (t, *J* = 7.9 Hz, 1H), 3.67 (s, 3H), 3.30 (m, 2H), 2.43-2.27 (m, 2H), 2.26-2.13 (m, 1H), 2.10-1.96 (m, 1H), 1.85-1.70 (m, 3H), 1.62 (m, 3H), 1.57

(d, J = 8.8 Hz, 1H), 1.46 (d, J = 7.9 Hz, 1H), 1.39 (s, 10H), 1.33-1.23 (m, 1H), 1.18-1.05 (m, 3H), 0.93 (d, J = 4.4 Hz, 1H), 0.88 (dd, J = 11.1, 6.8 Hz, 6H), 0.82 (s, 1H).

^{13}C NMR (126 MHz, $CDCl_3$) δ 179.52, 172.38, 171.56, 171.32, 155.50, 79.41, 59.68, 51.87, 50.52, 50.17, 40.08, 39.94, 38.11, 37.78, 33.40, 33.05, 32.02, 30.38, 27.82, 25.89, 25.70, 25.55, 18.72, 17.54.

LRMS (ESI) m/z: $[M+H]^+$ Found 539.0.

Methyl (S)-2-((S)-2-((S)-2-amino-3-methylbutanamido)-3-cyclohexylpropan amido)-3-((S)-2-oxopyrrolidin-3-yl)propanoate hydrochloride (6)

Compound 4 (0.54 g, 1.0 mmol) was dissolved in dry DCM (100 mL), then the 4 M HCl (2.5 mL, 10 mmol) was added slowly at 0 °C. The resulting mixture was stirred at ambient temperature for 12 h. Solvent was removed in vacuo and the crude product 5 was directly used in next step without further purification.

Methyl (2S)-2-((2S)-3-cyclohexyl-2-(3-methyl-2-(quinoline-2-carboxamido) butanamido) propanamido)-3-((S)-2-oxopyrrolidin-3-yl) propanoate (8d)

2-Quinolinecarboxylic acid (0.17 g, 1.0 mmol) was dissolved in 30 mL CH_2Cl_2 at -20 ℃, then the HATU (0.53 g, 1.4 mmol) was added. The solution was kept at -20 ℃ for 20 mins, and the intermediate 5 was added. After 30 mins later, the DIPEA (0.5 mL, 3.0 mmol) was added drop-wise, and the reaction mixture was stirred at -20 ℃ for 12 h. The resulting mixture was washed by 1 M HCl solution (100 mL×3), saturated $NaHCO_3$ solution (100 mL×3) and brine (100 mL×3). The organic phase layer was dried over Na_2SO_4 and concentrated in vacuo. The resulting mixture residue was purified by column chromatography (DCM: CH_3OH, 40: 1 v/v) to afford the pure product 7 (0.47 g, 80 %) as a light solid.

^{1}H NMR (500 MHz, $CDCl_3$) δ 8.73 (d, J = 9.3 Hz, 1H), 8.30 (d, J = 8.5 Hz, 1H), 8.25 (d, J = 8.5 Hz, 1H), 8.12 (d, J = 8.5 Hz, 1H), 7.85 (t, J = 8.3 Hz, 2H), 7.75 (ddd, J = 8.4, 6.9, 1.3 Hz, 1H), 7.61 (ddd, J = 8.1, 7.0, 1.1 Hz, 1H), 7.57 (d, J = 8.8 Hz, 1H), 7.08 (s, 1H), 4.69 (td, J = 9.3, 5.4 Hz, 1H), 4.60 (ddd, J = 11.8, 8.1, 3.5 Hz, 1H), 4.46 (dd, J = 9.2, 7.7 Hz, 1H), 3.70 (s, 3H), 3.42-3.31 (m, 2H), 2.48-2.31 (m, 3H), 2.30-2.22 (m, 3H), 1.91-1.79 (m, 2H), 1.71 (ddd, J = 13.6, 8.3, 5.3 Hz, 2H), 1.60 (d, J = 12.6 Hz, 1H), 1.44-1.37 (m, 1H), 1.33-1.25 (m, 1H), 1.05 (dd, J = 6.7, 3.6 Hz, 6H), 1.01-0.71 (m, 5H).

^{13}C NMR (126 MHz, $CDCl_3$) δ 179.46, 172.32, 171.60, 170.44, 164.33, 148.48, 146.04, 137.07, 129.73, 129.48, 128.91, 127.66, 127.18, 118.28, 58.92, 51.91, 50.79, 50.14, 40.13, 39.91, 37.83, 33.65, 33.16, 33.09, 31.90, 30.28, 27.51, 25.80, 25.69, 25.48, 18.99, 17.92.

LRMS (ESI) m/z: [M+H]+ Found 594.0.

N-(1-(((S)-3-cyclohexyl-1-(((S)-1-hydroxy-3-((S)-2-oxopyrrolidin-3-yl)propan-2-yl) amino)-1-oxopropan-2-yl)amino)-3-methyl-1-oxobutan-2-yl)quinoline-2-carboxamide (9d)

The synthesis of compound 8 is similar with the compound 3

^{1}H NMR (500 MHz, $CDCl_3$) δ 8.72 (d, J = 8.4 Hz, 1H), 8.30 (d, J = 8.4 Hz, 1H), 8.21 (d, J = 8.5 Hz, 1H), 8.02 (d, J = 8.5 Hz, 1H), 7.85 (d, J = 8.2 Hz, 1H), 7.76 (d, J = 8.4 Hz, 1H), 7.70 (ddd, J = 8.4, 6.9, 1.3 Hz, 1H), 7.65-7.56 (m, 2H), 6.76 (s, 1H), 4.61 (td, J = 9.6, 5.3 Hz, 1H), 4.50 (dd, J = 8.3, 6.9 Hz, 1H), 4.05 (td, J = 8.0, 4.1 Hz, 1H), 3.88 (s, 1H), 3.68-3.59 (m, 2H), 3.34-3.28 (m, 2H), 2.42-2.34 (m, 3H), 2.15-2.07 (m, 1H), 1.84-1.75 (m, 1H), 1.70 (m, 2H), 1.62-1.45 (m, 6H), 1.36-1.25 (m, 1H), 1.04 (d, J = 6.8 Hz, 6H), 1.02-0.96 (m, 3H), 0.84 (m, 2H).

^{13}C NMR (126 MHz, $CDCl_3$) δ 180.53, 172.88, 170.70, 164.65, 148.28, 145.94, 137.18, 129.69, 129.36, 128.91, 127.69, 127.20, 118.15, 65.22, 58.89, 51.41, 49.66, 40.18, 39.38, 37.90, 33.88, 33.16, 32.15, 31.77, 30.41, 27.89, 25.81, 25.70, 25.48, 19.09, 17.75.

LRMS (ESI) m/z: [M+H]+ Found 566.0.

N-(1-(((S)-3-cyclohexyl-1-oxo-1-(((S)-1-oxo-3-((S)-2-oxopyrrolidin-3-yl)propan-2-yl) amino)propan-2-yl)amino)-3-methyl-1-oxobutan-2-yl)-3-methylisoxazole-5-carbox amide (10a)

The synthesis of compound 10a is similar with the compound 10d

^{1}H NMR (600 MHz, $CDCl_3$) δ 9.49 (s, 1H), 8.27 (d, *J* = 6.7 Hz, 1H), 7.46 (d, *J* = 9.0 Hz, 1H), 7.42 (d, *J* = 8.4 Hz, 1H), 6.96 (s, 1H), 6.43 (s, 1H), 4.68 (m, 1H), 4.46 (dd, *J* = 8.7, 7.2 Hz, 1H), 4.39 (ddd, *J* = 10.6, 6.5, 4.4 Hz, 1H), 3.36 (t, *J* = 9.0 Hz, 1H), 3.31 (dd, *J* = 16.7, 9.0 Hz, 1H), 2.46 (s, 3H), 2.43 (d, *J* = 8.9 Hz, 1H), 2.36-2.31 (m, 1H), 2.22-2.17 (m, 1H), 2.04 (dd, *J* = 10.0, 5.2 Hz, 1H), 1.93-1.86 (m, 1H), 1.79 (dd, *J* = 12.2, 9.7 Hz, 1H), 1.73-1.65 (m, 3H), 1.63 (s, 1H), 1.54 (m, 3H), 1.14-1.06 (m, 3H), 0.95 (dd, *J* = 6.7, 2.2 Hz, 6H), 0.90-0.82 (m, 3H).

^{13}C NMR (151 MHz, $CDCl_3$) δ 199.51, 180.04, 173.44, 171.34, 170.56, 159.37, 158.33, 101.47, 58.66, 57.31, 51.16, 40.64, 40.21, 34.13, 33.46, 32.57, 31.15, 29.81, 29.68, 28.24, 26.33, 26.14, 25.99, 19.26, 18.19, 12.33.

HRMS (ESI) m/z: $[M+H]^+$ Calcd for C26H40N5O6: 518.2973; Found 518.2981.

N-(1-(((S)-3-cyclohexyl-1-oxo-1-(((S)-1-oxo-3-((S)-2-oxopyrrolidin-3-yl)propan-2-yl) amino)propan-2-yl)amino)-3-methyl-1-oxobutan-2-yl)-1H-indole-2-carboxamide (10b)

The synthesis of compound 10b is similar with the compound 10d

^{1}H NMR (500 MHz, DMSO-d_6) δ 11.56 (s, 1H), 9.40 (s, 1H), 8.44 (d, *J* = 7.8 Hz, 1H), 8.23 (d, *J* = 8.6 Hz, 1H), 8.13 (d, *J* = 7.7 Hz, 1H), 7.65-7.57 (m, 2H), 7.27 (dd, *J* = 2.8, 2.1 Hz, 1H), 7.17 (ddd, *J* = 8.2, 7.0, 1.1 Hz, 1H), 7.05-6.98 (m, 1H), 4.39-4.34 (m, 2H), 4.21 (ddd, *J* = 11.6, 7.8, 3.9 Hz, 1H), 3.15 (t, *J* = 9.2 Hz, 1H), 3.08-3.01 (m, 1H), 2.34 -2.19 (m, 1H), 2.12 (m, 2H), 1.90 (m, 1H), 1.72 (m, 1H), 1.68-1.57 (m, 5H), 1.55-1.40 (m, 4H), 1.08 (m, 3H), 0.92 (dd, *J* = 6.8, 2.6 Hz, 6H), 0.89-0.75 (m, 3H).

^{13}C NMR (126 MHz, DMSO-d_6) δ 200.64, 178.29, 172.58, 170.87, 160.98, 136.49, 131.26, 126.98, 123.38, 121.50, 119.68, 112.23, 103.71, 58.27, 56.12, 50.46, 37.16, 33.45, 32.92, 32.05, 30.32, 29.26, 27.21, 25.98, 25.71, 25.54, 19.14, 18.64.

HRMS (ESI) m/z: $[M+H]^+$ Calcd for $C_{30}H_{41}N_5O_5$ 552.318; Found 552.3187.

N-(1-(((S)-3-cyclohexyl-1-oxo-1-(((S)-1-oxo-3-((S)-2-oxopyrrolidin-3-yl)propan-2-yl)amino)propan-2-yl)amino)-3-methyl-1-oxobutan-2-yl)benzofuran-2-carboxamide（10c）

The synthesis of compound 10c is similar with the compound 10d

^{1}H NMR (600 MHz, $CDCl_3$) δ 9.53 (s, 1H), 8.27 (d, *J* = 7.0 Hz, 1H), 7.91 (d, *J* = 8.4 Hz, 1H), 7.64 (d, *J* = 7.8 Hz, 1H), 7.51 (s, 1H), 7.48 (d, *J* = 8.4 Hz, 1H), 7.43 (d, *J* = 8.9 Hz, 1H), 7.38 (dd, *J* = 11.5, 4.2 Hz, 1H), 7.28-7.24 (m, 1H), 7.18 (s, 1H), 4.72 (dd, *J* = 14.9, 8.6 Hz, 1H), 4.66-4.60 (m, 1H), 4.50-4.44 (m, 1H), 3.36 (t, *J* = 9.0 Hz, 1H), 3.31-3.27 (m, 1H), 2.46 (dt, *J* = 14.9, 7.5 Hz, 1H), 2.32 (dd, *J* = 10.7, 5.1 Hz, 1H), 2.22 (dd, *J* = 13.8, 6.9 Hz, 1H), 2.11-2.03 (m, 1H), 1.93-1.87 (m, 1H), 1.83-1.77 (m, 1H), 1.76 – 1.66 (m, 3H), 1.58-1.53 (m, 3H), 1.49 (m, 1H), 1.34-1.29 (m, 1H), 1.07-1.02 (m, 2H), 1.00 (dd, *J* = 6.7, 3.0 Hz, 6H), 0.92-0.78 (m, 3H).

^{13}C NMR (151 MHz, $CDCl_3$) δ 199.44, 180.06, 173.45, 170.98, 158.89, 154.86, 148.15, 127.45, 127.11, 123.76, 122.72, 111.90, 111.05, 58.44, 57.12, 51.38, 40.64, 40.21, 37.93, 34.18, 33.40, 32.64, 31.52, 29.96, 28.10, 26.28, 26.09, 25.96, 19.29, 18.46.

HRMS (ESI) m/z: $[M+H]^+$ Calcd for $C_{30}H_{41}N_4O_6$: 553.3021; Found 553.3035.

N-(1-(((S)-3-cyclohexyl-1-oxo-1-(((S)-1-oxo-3-((S)-2-oxopyrrolidin-3-yl)propan-2-yl)amino)propan-2-yl)amino)-3-methyl-1-oxobutan-2-yl)quinoline-2-carboxamide (10d)

To a solution of the 8 (0.34 g, 0.6 mmol) in CH_2Cl_2, DMP (0.3 g, 0.7 mmol) was added slowly and the reaction mixture was stirred at room temperature for 5 h. The completion of the reaction was confirmed by TLC then quench and concentrate the reaction, and the

reaction was filtered and washed with saturated NaHCO3 solution (50 mL×3) and brine (50 mL×3). The organic phase was dried over $MgSO_4$ and concentrated, and the residue was purified by column chromatography (DCM: CH_3OH, 20: 1 v/v) to afford the pure product 1 (0.24 g, 71 %) as a light solid.

^{1}H NMR (500 MHz, $CDCl_3$) δ 9.54 (s, 1H), 8.73 (d, J = 8.8 Hz, 1H), 8.30 (d, J = 8.5 Hz, 1H), 8.23 (d, J = 8.5 Hz, 1H), 8.21 (d, J = 7.1 Hz, 1H), 8.06 (d, J = 8.5 Hz, 1H), 7.86 (d, J = 7.9 Hz, 1H), 7.74-7.70 (m, 1H), 7.62-7.58 (m, 1H), 7.53 (d, J = 8.4 Hz, 1H), 6.96 (s, 1H), 4.71 (m, 1H), 4.52-4.42 (m, 2H), 3.35 (m 2H), 2.50-2.35 (m, 4H), 2.11-2.03 (m, 1H), 1.95-1.88 (m, 1H), 1.81 (dd, J = 11.9, 9.3 Hz, 1H), 1.75-1.70 (m, 2H), 1.62 (d, J = 12.6 Hz, 1H), 1.55 (dd, J = 12.0, 7.6 Hz, 3H), 1.45 (d, J = 3.4 Hz, 1H), 1.05 (d, J = 6.8 Hz, 6H), 1.00 (d, J = 7.7 Hz, 2H), 0.90-0.80 (m, 3H).

^{13}C NMR (126 MHz, $CDCl_3$) δ 199.21, 179.61, 173.07, 170.62, 164.46, 148.40, 145.97, 137.13, 129.72, 129.39, 128.91, 127.68, 127.20, 118.21, 58.77, 56.64, 50.94, 40.15, 39.55, 33.79, 33.09, 31.88, 30.36, 29.62, 29.21, 27.76, 25.79, 25.67, 25.46, 19.08, 17.75.

HRMS (ESI) m/z: $[M+H]^+$ Calcd for $C_{31}H_{42}N_5O_5$: 564.318; Found 564.3193.

```
Conservation:  99   999   99 9999999   999  99 9    9 99           9 9    9    9 9
GI.1        1  APPTLWSRVTKFGSGWGFWVSPTVFITTTHVVPTGVKEFFGEPLSSIAIHQAGEFTQFRFSKKMRPDLTG   70
GII.4       1  APPSIWSRIVNFGSGWGFWVSPSLFITSTHVIPQGAKEFFGVPIKQIQVHKSGEFCRLRFPKPIRTDVTG   70
GV          1  APVSIWSRVVQFGTGWGFWVSGHVFITAKHVAPPKGTEIFGRKPGDFTVTSSGDFLKYYFTSAVRPDIPA   70
Consensus_ss:      hhhhh       eeeee eeeeeeee      hhhh   hhheeeee   eeeee          e

Conservation:  9 99 9  99 9   9 99  99   99 999  9   9    9 99 999999 999  9999 99999
GI.1       71  MVLEEGCPEGTVCSVLIKRDSGELLPLAVRMGAIASMRIQGRLVHGQSGMLLTGANAKGMDLGTIPGDCG  140
GII.4      71  MILEEGAPEGTVVTLLIKRPTGELMPLAARMGTHATMKIQGRTVGGQMGMLLTGSNAKSMDLGTTPGDCG  140
GV         71  MVLENGCQEGVVASVLVKRASGEMLALAVRMGSQAAIKIGSAVVHGQTGMLLTGSNAKAQDLGTIPGDCG  140
Consensus_ss:  eee        eeeeeee     eeeeeee   eeeeeeeeeee   eeeeee

Conservation:       99  9 99  99 999 99   9999
GI.1          141  APYVHKRGNDWVVCGVHAAATKSGNTVVC  169
GII.4         141  CPYIYKRGNDYVVIGVHTAAARGGNTVIC  169
GV            141  CPYVYKKGNTWVVIGVHVAATRSGNTVIA  169
Consensus_ss:        eeeee  eeeeeeeeeee     eee
```

Figure S1: Amino acid sequence alignment of norovirus proteases.

Amino acid sequence alignment of a HuNoV GI.1 (M87661), HuNoV GII.4 (DQ658413) and GV (DQ285629) proteases. In the first line, the conservation indices of 9 indicates highly conserved amino acids. According to the PSIPRED secondary structure predictions sequences in bold are part of alpha-helixes and underlined sequences are β-strands. The consensus-predicted secondary structure is shown in the last line of each block. If the fraction of helix or strand predictions among representative sequences in a position is greater than 0.5, the consensus letter is 'h' or 'e', respectively. Alignment was made with the PROMALS server, acquired mutations are highlighted.

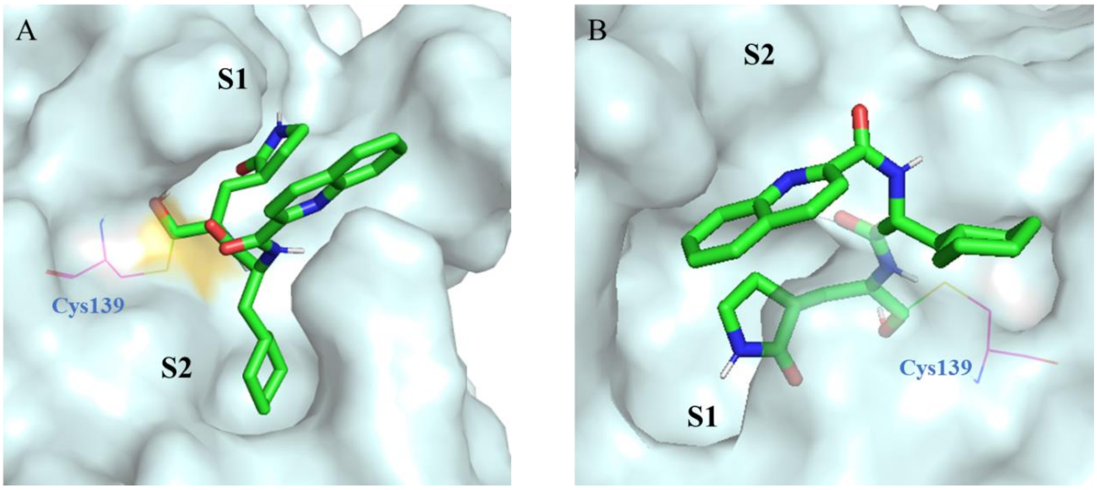

Figure S2: Molecular docking was performed to study the potential interactions between compound 4 with the GI.1 and GII.4 norovirus 3CLpro.

The PDB file of the protein was obtained from protein data bank using the code 3UR9 (GI.1 norovirus 3CLpro) and 6NIR (GII.4 norovirus 3CLpro). The Schrödinger program was used for calculations and PyMol program for visualizations.
